# The Effect of Final Rinse Agitation with Ultrasonic or 808 nm Diode Laser on Coronal Microleakage of Root-canal Treated Teeth

**DOI:** 10.22037/iej.v13i1.17248

**Published:** 2018

**Authors:** Mohsen Ramazani, Mohammad Asnaashari, Roghayyeh Ahmadi, Nafiseh Zarenejad, Alireza Rafie, Jamshid Yazadani Charati

**Affiliations:** a *Department of Endodontics, Sari Dental School, Mazandaran University of Medical Sciences; *; b *Iranian Center For Endodontic Research , Research Institute of Dental Research, Department of Endodontics, Dental School, Shahid Beheshti University of Medical Sciences, Tehran, Iran; *; c *Dentist, Sari, Iran; *; d *Restorative Dentistry, Sari Dental School, Mazandaran University of Medical Sciences, Sari, Iran; *; e *Department of Immunology, Molecular and Cell Biology Research Center, Mazandaran University of Medical Sciences, Sari, Iran; *; f *Biostatistic Department, School of Health, Mazandaran University of Medical Sciences, Sari, Iran*

**Keywords:** Apical Seal, Diode Laser, Irrigant Agitation, Irrigant Solution, Diode Laser, Ultrasonic

## Abstract

**Introduction:**

This *in vitro* study aimed at comparing the effect of agitating the final irrigant solutions of root canal by ultrasonic or using 808 nm Diode laser on the apical seal of canal.

**Methods and Materials:**

A total of 90 extracted human maxillary central incisors were prepared up to size #45 and were randomly assigned to 4 experimental groups (*n*=20) and two control groups (*n*=5) respectively, as follows: *I*): 3 mL of 5.25% NaOCl was agitated as final irrigant solution with ultrasonic for 30 sec. The ultrasonic tip was 1 mm shorter than the working length, *II*): 3 mL of 5.25% NaOCl was agitated as final irrigant with 808 nm Diode laser for 30 sec. Fiber tip, placed in 1 mm shorter from working length was spirally moved coronally, *III*): 3 mL of 17% EDTA was agitated as final irrigant with 808 nm Diode laser for 30 sec and was applied similar to group II, *IV*): 3 mL of 17% EDTA was stimulated as final irrigant with ultrasonic for 30 sec and was applied similar to I. Apical seal was assessed by Dual Chamber technique using Bovine Serum Albumin protein. Kruskal-Wallis and Mann Whitney tests were used with significance level lower than 0.05% for statistical analysis.

**Results:**

The average leakage in the negative control, positive control, and groups I, II, III, IV were: 0.00, 13.5±5.1, 1.72±2.9, 5.12±5.6, 3.36±3.7, 2.4±4.2, respectively. Statistical analysis showed significant difference between groups (*P*<0.05). There was a significant difference between groups 1 and 2 in terms of protein leakage**. **

**Conclusion:**

Agitating 5.25% sodium hypochlorite solution as the final irrigant with ultrasonic is more effective in apical leakage reduction compared to other groups.

## Introduction

Root canal therapy is a treatment through which the patient is able to hold his natural teeth with maintaining its performance and beauty [1]. Success in endodontics is primarily determined based on three factors of cleaning and shaping, full disinfection and filling [2]. Root canal therapy is performed with the aim of removing pathogens and their products, to fill canal space with a three-dimensional filling and make a proper apical seal [3]. Given the importance of disinfecting the canal as a prerequisite for successful root canal therapy, mechanical instrumentation and canal irrigation have an important role in the process of root canal therapy [2]. Among the existing irrigant solutions, sodium hypochlorite and ethylene diamine-tetra-acetic acid (EDTA) are commonly used [4]. Studies have shown that the use of these solutions has a significant effect after the end of canal cleaning and shaping as final irrigant solutions before filling the root canal in removal of smear layer [4, 5]. Microorganisms remain despite the process of root canal cleaning and shaping, due to its complex morphology including isthmii, deltas and lateral canals which act as a place for bacteria, debris and necrotic tissues to harbor [5]. Regardless of the instrumentation techniques, 35% or more of the root canal surfaces remain unprepared [6]. Accordingly, the irrigants ability to penetrate these areas is important for debridement and disinfecting root canal [4]. These solutions should be in direct contact with the root canal wall surface to improve effectiveness [4]. However, reaching the apical part is usually difficult for the irrigants due to small diameter of canals. The traditional irrigation with the syringes are widely used in endodontic therapy, but the studies have shown that the effect of irrigants is directly associated with the needle penetration depth on the apical region and this is not possible in narrow or curved canals. That^’^s why; some techniques and tools such as ultrasonics and laser are applied to improve the effectiveness of irrigants [7-11], because they render the irrigants to reach the apical third of the root canal system. Passive Ultrasonic Irrigation (PUI) is described as using an activated file with ultrasonic in passive mode to create acoustic streaming. PUI is able to remove endodontics biofilm and help irrigants to penetrate across canal walls more effectively. Using a combination of PUI with NaOCl removes more debris than irrigation with a syringe [7]. In the last two decades, laser technology has gained the spotlight as an adjunct treatment in endodontics [12]. Lasers operate in continuous wave or pulse mode or by the use of an optical fiber conductor. Specific wavelengths of laser light are capable of penetrating deep into the dentinal tubules and eliminating the microorganisms, removing the smear layer or changing the surface morphology of dentin. Diode lasers have been proposed for disinfection of the root canal due to their optimal antibacterial properties and low cost in relation to most laser used in endodontics. It has been demonstrated that *E. faecalis *can be largely or completely eliminated by the application of diode laser alone or in combination with irrigating solutions. The available diode laser wavelengths for dental application range from 800 to 1064 nm. Some previous studies have assessed the efficacy of 810 nm and 980 nm lasers separately and their results confirm their acceptable antimicrobial efficacy. Laser-activated irrigation increases its efficiency through cavitation, subsequent bubbles collapse and creating acoustic streaming, which leads to removal of smear layer and debris from the canal wall [9, 13]. According to the aforementioned items, this study aimed to evaluate the efficacy of the final rinse agitation with ultrasonic or 808 nm diode laser on apical seal of human single-rooted teeth.

## Materials and Methods

In this *in vitro* study, 90 human maxillary incisors extracted due to periodontal reasons were collected. The teeth were held in sodium hypochlorite (5.25%) (Golrang, Pakshoo Co. Tehran Iran) for 24 h to remove any debris and pollution and later external root surfaces were cleaned using sterile ultrasonic inserts (Varios, NSK, Nakanishi Inc., Tokyo, Japan) to take dental plaque and organic tissues. The samples were then stored in normal saline. The selected teeth clinically had straight roots without any anatomical anomaly. All samples were subjected to buccolingual and mesiodistal radiography. Calcification, internal or external resorption or cracks (visible under the light of stereo microscope), severe decay of dental crown and roots, large restoration of crown, root fractures and severe curvature of the root, severe depression on the root surface and apical foramen larger than File #40, were considered as exclusion criteria. 

The crown of all samples was cut from cemento-enamel junction with a fissure bur (Jota, Ruthi, Switzerland) under copious water. Then using the K-File #15 (Dentsply Maillefer, Ballaigues, Switzerland) roots length were determined. For this purpose, the file was placed in the root so that its tip could be observed from apical foramen. After calculating the roots length, the working length was considered as 1 mm shorter. The length of all samples were the same and equal to 13 mm. The coronal portion of the roots was prepared by crown down technique using #2 and 3 Gates Glidden drills (Dentsply Maillefer, Ballaigues, Switzerland). Then apical portion of the roots were prepared up to the #45 file (as Master Apical File). During sample preparation and the cleaning and shaping stages, normal saline was used for irrigation. All canals were prepared by the standard step back method and were kept in normal saline. Based upon simple allocation protocol, the samples were randomly divided into 6 Groups of 4 experimental groups (*n*=20) and two control groups (*n*=5). After randomized assignation in the groups, the interventions were applied in accordance with the following procedure:

*Group 1*: The use of 3 mL of 5.25% NaOCl as a final irrigant solution which was transferred to canals with a syringe and activated with ultrasonic set (Nakanishi Inc., Tokyo, Japan) and the tip of E8 for 30 sec (That is, three times every 10 sec and 1 mL of the solution, and 10-sec-intervals to avert from temperature increase).

*Group* *2* *:* The use of 3 mL of 5.25% NaOCl similar to (Group I) as final irrigant solution radiated by 808 nm diode laser with the power of 1.5 W and continuous mode with 200 micron fiber tip and the fiber tip was placed in 1 mm shorter of the working length (for better contact with the entire surface of the wall this technique is used due to direct radiation of LASER beam) and was helically moved from apical to the coronal portion. Each time irradiation lasts for 10 sec. It was moved from apical to the coronal with the speed of 1.2 mm/s. The total number of irradiation times for each canal is 3 times at intervals of 10 sec.

*Group* *3*: The use of 3 mL 17% EDTA (similar to Groups 1 and 2) (Diadent Inc., Chongchong Buch Do, Korea) as a final irrigant solution irradiated by 808 nm diode laser with the power of 1.5 W and continuous mode with the fiber tip of 200 μm that the fiber tip was placed in 1 mm shorter of working length (for better contact with the entire surface of the wall this technique is used due to direct radiation of laser beam) and was helically moved from apical portion to the coronal portion. Each time irradiation lasts for 10 sec. The total number of irradiation times was 3 times at intervals of 10 sec to avoid temperature increase.

*Group 4*: The use of 3 mL (similar to Groups 1, 2, 3) of %17 EDTA as the final irrigant solution which was transferred to canals by syringe and was agitated for 30 sec with ultrasonic and E8 tip for 30 sec (that is, three times each time 10 sec and 1 mL of solution at intervals of 10 sec to avoid temperature increase). In all groups, the volume of the used solution and the time of agitation are equal. Positive control samples were prepared without agitation and filling was not done. The five negative control samples were prepared and all canals were filled with wax. 

After this stage, the canals were dried with paper cone (AriaDent, Tehran, Iran) and obturated with gutta-percha 2% (Diadent Inc., Chongchong Buch Do, Korea) and AH-26 sealer (Dentsply, Tulsa Dental, Tulsa, OK, USA) using lateral Cold Condensation technique using the spreader (Dentsply Maillefer, Ballaigues, Switzerland) to be ready for the next steps. Outer surface of root was covered using two layers of varnish (Nail Polish) except for 2 mm apical zone. No coverage was applied for positive control group samples, whereas in negative group, the outer surfaces was completely covered with two layers of varnish.

All the samples were mounted in a dual-chamber leakage apparatus. First, the teeth were inserted from the cap end of a 1.5-mL plastic Eppendorf cylinder (Elkay, Shrewbury, MA, USA). The ending 3 mm of cylinders were cut previously, so the root tips passed through this part and were visible. Interfaces between the tubes and the teeth were sealed with sticky wax. The cylinders were placed in pre-autoclaved 10-mL glass vial tube with identical dimensions. The glass vials had been filled previously with 9 mL of distilled water. The junction line between microtube and vial was tightly covered and sealed with Parafilm (Supa Co., Tehran, Iran). The whole system was sterilized with ethylene oxide gas for 12 h. The plastic cylinders were filled with 1 mL of 1% bovine serum albumin (BSA, Sigma-Aldrich, St. Louis, MO, USA). The assembly was incubated again at 37^°^C and a relative humidity of 90% for 30 days (test period). BSA was refreshed every day throughout the experiment. Bradford indicator was used to measure the concentration of leaked albumin form the upper chamber into the lower one at the end of the 30^th^ day. Bradford protein reagent is an aqueous solution of Coomassie Brilliant Blue G (Sigma-Aldrich, St. Louis, MO, USA), ethanol, and phosphoric acid. The procedure is based on the formation of a complex between the dye, Brilliant Blue G, and proteins in solution. According to the manufacturer, albumin leakage into the solution and subsequent formation of the protein-dye complex would shift the wavelength of maximum absorption of Coomassie Brilliant Blue G from 465 to 596 nm. Color development is rapid. Only 5-min incubation is sufficient to read the samples at 595 nm. The amount of absorption is proportional to the protein present. The glass tubes were separated. Then 50 μL of test solution of the vials was pipetted into a new Eppendorf tube and 150 microliters of Bradford protein reagent was added to the tube, and the contents were mixed using spectrophotometry, and the maximum absorption was measured to calculate the microleakage. Statistical analysis of the data was conducted using the Kruskal-Wallis followed by the Mann-Whitney tests. The level of significance was set at 0.05 (SPSS software for Windows, SPSS version 23.0, SPSS Chicago, IL, USA).

## Results

**Figure1:**
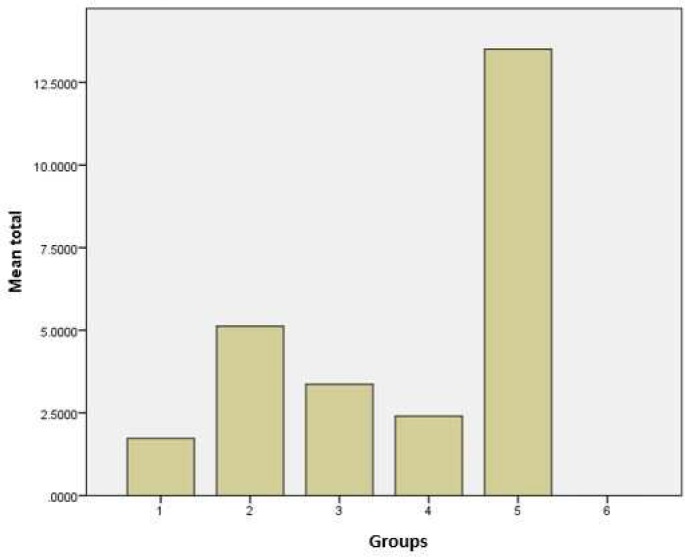
Graphical representation of protein leakage in experimental groups

Average leakage in the negative control samples, positive and groups 1, 2, 3 and 4 was 0.00, 13.5±5.1, 1.72±2.9, 5.12±5.6, 3.36±3.7, 2.4±24.2 respectively. Statistical analysis showed a significant difference among the groups (*P*<0.05). According to the statistical analysis, no leakage was observed in negative control group and the most leakage was found in the positive control group (Figure 1). In the comparison between groups 1 and 4, sodium hypochlorite and EDTA had been agitated with ultrasonic at the same time and volume. There was no significant difference among the effects of solutions on the apical seal (*P*>0.05). In the comparison between groups 2 and 3, sodium hypochlorite and EDTA with 808 nm laser diode had been agitated at the same time and volume. No significant difference was observed between the leakages of protein in these two groups (*P*>0.05). In a comparison between the groups 1 and 2 in which the sodium hypochlorite solution had been activated with ultrasonic and 808 nm laser diode, the results have shown significant differences between the two groups (*P*<0.05). In a comparison between groups 3 and 4, where EDTA solution had been activated by ultrasonic and 808 nm laser diode, significant difference was not observed between the groups (*P*>0.05). Among the four experimental groups, the first group (sodium hypochlorite agitated with ultrasonic) showed the least amount of protein leakage (*P*<0.05).

## Discussion

Removal of necrotic pulp tissues, dentinal debris, the microorganisms remained from root canal is a prerequisite for success in endodontic treatment [14]. Mechanical cleaning of canals significantly reduces microorganisms inside the canal, but does not sterile the canal [2, 13]. To improve the mechanical method of preparation, antimicrobial irrigant solutions have been recommended. The main function of irrigants is cleaning the root canal in the process of shaping and enlargement. Commonly used irrigant solutions in endodontics include sodium hypochlorite (NaOCl) and ethylene diamine tetra-acetic acid (EDTA) that are very beneficial through the process of cleaning and shaping as well as irrigation [15, 16]. For example, the advantages of sodium hypochlorite during preparation can be improving the cutting effect of hand tools, reducing the torque on the rotary NiTi files. Moreover, the use of these solutions as the final irrigant solutions effectively helps removal of the smear layer from the root canal walls [17]. On the other hand, one of the problems of sodium hypochlorite is having high surface tension that restricts its penetration into the dentinal tubules and the root canal irregularities [18]. Studies have shown that the use of syringe is not effective for irrigation in the apical third of canal. This traditional technique distributes the solution in 0.1-1 mm beyond the tip of the syringe because canal irrigants must be in contact with the canal to improve the effect of this irrigation in the apical region [10]. Available ways to improve the effectiveness of irrigating solutions include change of concentration, volume, pH, temperature and conditions of maintenance and agitation [14, 16]. Among the tools used to agitate the irrigant solutions can be ultrasonic exposure. Ultrasonic influence is defined by its ability in the formation of bubbles called cavitation and acoustic streaming [19]. Laser is another way which is used for this purpose. With the advent of wide applications of lasers in dentistry, it could have attracted the clinicians’ attention toward the use of it in endodontics in order to agitate irrigant solutions for improving their effectiveness [20]. 

In the recent years, application of laser technology in clinical dentistry has considerably increased, mainly due to introduction of different laser wavelength, methods and delivery systems. Laser therapy is known as an efficient modality in endodontic treatment due to multiple advantages such as smear layer removal, decreasing the bacterial count and reducing the apical microleakage. Studies have shown that differences in the wavelength, power, irradiation, time, spot size and number of cycles are responsible for the variable efficacy of lasers reported. Laser-based mechanism in enabling solutions is according to absorption of laser energy, the formation of bubbles, the collapse of these bubbles and creating acoustic streaming [9]. Laser diode due to less space occupied by the device, low cost, thin and elastic fiber tip (which enables practitioners to use it in narrow and curved canals) has been very important. Diode laser, which is generally, introduced in endodontic covers wave lengths in the range of 800-980 nm [21]. It has a high absorption in pigments, but its absorption in water and hydroxyapatite are low. Most bacteria produce pigments and also due to the penetration depth of bacteria up to 1100 microns within the dentinal tubules, diode laser can be a good choice for use in removing and reducing bacteria because it has passed hydroxyapatite and has a high penetration depth. 

Microbiological studies have shown that Nd: YAG laser gives the best results in reducing microorganisms even to a depth of 1000 µm [22]. Diode laser with a wavelength of 810 nm ranks second in terms of reducing microorganisms (about 63%) [9, 19]. This study aimed to compare the effect of agitating final irrigant solutions with 808 nm diode laser and ultrasonic waves on root canal apical seal. According to the results obtained in this study, sodium hypochlorite agitation with ultrasonic waves is effective in reducing the apical leakage. The studies carried out in this regard confirm the above results including a study conducted in 2015 by Kanumuru *et al.* [23] for comparing the penetration of different solutions agitated by different methods. 

The results of the study showed that ultrasonic and reciprocating groups led to better penetration of solutions. In another study which was done by Justo *et al.* [24], the effect of final irrigation on removing debris was studied. Different solutions such as sodium hypochlorite, chlorhexidine and saline were considered as the main groups

The results showed that in groups that ultrasonic was performed; removing debris from the apical third of the root was done more effectively. And it was also observed that the type of used solution is not effective on removing debris. In the present study, comparison between the groups activated with the same tools, showed that the type of solution used is not effective in reducing protein leakage.

The sealing ability properties of endodontic filling materials are investigated using various methods including dye penetration, clearing techniques, radioisotopes, marginal adaptation, bacterial leakage, *etc.* [25]. A series of advantages and disadvantages have been mentioned for each of these techniques, for example, bacterial leakage evaluates sealing in all assessed areas while only adjacent walls of materials are evaluated in dye leakage [26]. The bacterial leakage studies focus on the main pathogenic factor, but even this technique may not reflect actual clinical conditions because the specific species are used with a limited number of bacteria and therefore the synergistic bacterial effect, environmental influence, temperature changes, salivary enzymes and buffering antibody substances are ignored. In this study, protein leakage method has been used to evaluate the sealing ability some of the studies that used this technique include the studies by Saghiri *et al.* in 2008 [27], Vosoughhosseini *et al.* in 2011 [28] and Zarenejad *et al.* in 2015 [25].

In a study carried out by Wang *et al. *[16], the effect of 980 nm diode laser irradiation was investigated on apical leakage after canal obturation. The samples were irradiated with a 980 nm diode laser and the apical leakage was evaluated using a dye penetration. The results showed that 980 nm diode laser is useful to reduce apical leakage. However, in this study the effect of agitating the solution was investigated by 880 nm diode laser on apical seal using protein leakage method [16]. In 2016, Ayranci *et al.* [17] examined the effect of agitating irrigant solutions with laser and ultrasonic on removing the smear layer on the apical third and middle one-third of roots. In this study, Er: YAG was used with a wavelength of 2940 nm. The results showed that laser agitated irrigant combined of 17% EDTA and 5.25% NaOCl each for 30 sec is the most effective on removing the smear layer in the apical third [17]. In that study, the tip of laser probe was placed in pulp chamber and the ultrasonic tip was placed in 1 mm shorter than the working length. The results showed that the agitating combination of these two solutions with ultrasonic, removes more smear layer compared to the groups in which sodium hypochlorite had been agitated only with ultrasonic and laser.

In contrast, the results obtained from comparing groups 1 and 2 in our study suggest more effectiveness of sodium hypochlorite agitation with ultrasonic waves compared to that of laser on apical seal (*P*<0.05). Different results may be due to different types of laser used in this experiment (wavelength, power). Moreover, the results showed that there is no significant difference in reducing leakage between agitating the solutions, EDTA and NaOCl with similar tools (*P*>0.05) (Comparing between groups 1-2 and 3-4). The study results showed that ultrasonic agitation of these two solutions is more effective than NaOCl agitation with laser. This means that the simultaneous use of the two solutions can be considered as a factor in removing the smear layer [23]. In a study conducted by Arslan in 2013 [29], the 808 nm diode laser agitating effect 15% EDTA as the final irrigant solution of canal began at different times. In that study, the number of open tubules in the apical third was examined after the test using electron microscopy. The results showed that agitation of 15% EDTA with an 808 nm diode laser for 20 sec has been effective as a final irrigant solution to remove the smear layer on the apical third and the most disruption in the dentinal tubules was observed. That study aimed to compare the agitation effect of 15% EDTA by laser in different times and activating the solution with ultrasonic waves has not investigated. Moreover, it is necessary to mention that the volume used from the solution was 15 mL all of which can be the reasons for different results. The possible reason for the lack of effect of agitating sodium hypochlorite with diode laser on the apical seal can be attributed to cross-reactivity between them and the conditions provided during the study (time of agitation, the volume of the solution, device settings) and the amount of laser energy absorbed by the solution. 

## Conclusion

It is concluded that strengthening final irrigant solution (5.25% NaOCl) with ultrasonic waves is a good way to minimize the protein leakage in root canal treatment processes.

## References

[1] Hoskin E, Veitz-Keenan A (2016). Antibiotics are not useful to reduce pain associated with irreversible pulpitis. Evid Based Dent.

[2] Del Fabbro M, Corbella S, Sequeira-Byron P, Tsesis I, Rosen E, Lolato A, Taschieri S (2016). Endodontic procedures for retreatment of periapical lesions. Cochrane Database Syst Rev.

[3] Jain P, Yeluri R, Garg N, Mayall S, Rallan M, Gupta S, Pathivada L (2015). A Comparative Evaluation of the Effectiveness of Three Different Irrigating Solution on Microorganisms in the Root Canal: An Invivo Study. J Clin Diagn Res.

[4] Guneser MB, Akbulut MB, Eldeniz AU (2016). Antibacterial effect of chlorhexidine-cetrimide combination, Salvia officinalis plant extract and octenidine in comparison with conventional endodontic irrigants. Dent Mater J.

[5] Estrela C, Bueno MR, Couto GS, Rabelo LE, Alencar AH, Silva RG, Pecora JD, Sousa-Neto MD (2015). Study of Root Canal Anatomy in Human Permanent Teeth in A Subpopulation of Brazil's Center Region Using Cone-Beam Computed Tomography - Part 1. Braz Dent J.

[6] Agrawal V, Kapoor S, Agrawal I Critical Review on Eliminating Endodontic Dental Infections Using Herbal Products. J Diet Suppl.

[7] Khalap ND, Kokate S, Hegde V (2016). Ultrasonic versus sonic activation of the final irrigant in root canals instrumented with rotary/reciprocating files: An in-vitro scanning electron microscopy analysis. J Conserv Dent.

[8] Camacho-Alonso F, Salmeron-Lozano P, Martinez-Beneyto Y (2017). Effects of photodynamic therapy, 2 % chlorhexidine, triantibiotic mixture, propolis and ozone on root canals experimentally infected with Enterococcus faecalis: an in vitro study. Odontology.

[9] Elnaghy AM, Elsaka SE (2017). Effect of sodium hypochlorite and saline on cyclic fatigue resistance of WaveOne Gold and Reciproc reciprocating instruments. Int Endod J.

[10] Plotino G, Cortese T, Grande NM, Leonardi DP, Di Giorgio G, Testarelli L, Gambarini G (2016). New Technologies to Improve Root Canal Disinfection. Braz Dent J.

[11] Mondet J, Hussein K, Mossuz P (2015). Circulating Cytokine Levels as Markers of Inflammation in Philadelphia Negative Myeloproliferative Neoplasms: Diagnostic and Prognostic Interest. Mediators Inflamm.

[12] Sohrabi K, Sooratgar A, Zolfagharnasab K, Kharazifard MJ, Afkhami F (2016). Antibacterial Activity of Diode Laser and Sodium Hypochlorite in Enterococcus Faecalis-Contaminated Root Canals. Iran Endod J.

[13] Eymirli A, Nagas E, Uyanik MO, Cehreli ZC (2017). Effect of Laser-Activated Irrigation with Ethylene Diaminetetraacetic Acid and Phytic Acid on the Removal of Calcium Hydroxide and Triple Antibiotic Paste from Root Dentin. Photomed Laser Surg.

[14] Camacho-Alonso F, Salmeron-Lozano P, Martinez-Beneyto Y (2016). Effects of photodynamic therapy, 2 % chlorhexidine, triantibiotic mixture, propolis and ozone on root canals experimentally infected with Enterococcus faecalis: an in vitro study. Odontology.

[15] Samiei M, Shahi S, Abdollahi AA, Eskandarinezhad M, Negahdari R, Pakseresht Z (2016). The Antibacterial Efficacy of Photo-Activated Disinfection, Chlorhexidine and Sodium Hypochlorite in Infected Root Canals: An in Vitro Study. Iran Endod J.

[16] Wang X, Sun Y, Kimura Y, Kinoshita J, Ishizaki NT, Matsumoto K (2005). Effects of diode laser irradiation on smear layer removal from root canal walls and apical leakage after obturation. Photomed Laser Surg.

[17] Ayranci LB, Arslan H, Akcay M, Capar ID, Gok T, Saygili G (2016). Effectiveness of laser-assisted irrigation and passive ultrasonic irrigation techniques on smear layer removal in middle and apical thirds. Scanning.

[18] Elnaghy AM, Elsaka SE (2016). Effect of sodium hypochlorite and saline on cyclic fatigue resistance of WaveOne Gold and Reciproc reciprocating instruments. Int Endod J.

[19] Keles A, Kamalak A, Keskin C, Akcay M, Uzun I (2016). The efficacy of laser, ultrasound and self-adjustable file in removing smear layer debris from oval root canals following retreatment: A scanning electron microscopy study. Aust Endod J.

[20] Eymirli A, Nagas E, Uyanik MO, Cehreli ZC (2016). Effect of Laser-Activated Irrigation with Ethylene Diaminetetraacetic Acid and Phytic Acid on the Removal of Calcium Hydroxide and Triple Antibiotic Paste from Root Dentin. Photomed Laser Surg.

[21] Lagemann M, George R, Chai L, Walsh LJ (2014). Activation of ethylenediaminetetraacetic acid by a 940 nm diode laser for enhanced removal of smear layer. Aust Endod J.

[22] Bago Juric I, Plecko V, Anic I, Plesko S, Jakovljevic S, Rocca JP, Medioni E (2016). Antimicrobial efficacy of photodynamic therapy, Nd:YAG laser and QMiX solution against Enterococcus faecalis biofilm. Photodiagnosis Photodyn Ther.

[23] Kanumuru PK, Sooraparaju SG, Konda KR, Nujella SK, Reddy BK, Penigalapati SR (2015). Comparison of Penetration of Irrigant Activated by Traditional Methods with A Novel Technique. J Clin Diagn Res.

[24] Martins Justo A, Abreu da Rosa R, Santini MF, Cardoso Ferreira MB, Pereira JR, Hungaro Duarte MA, Reis So MV (2014). Effectiveness of final irrigant protocols for debris removal from simulated canal irregularities. J Endod.

[25] Zarenejad N, Asgary S, Ramazani N, Haghshenas MR, Rafiei A, Ramazani M (2015). Coronal microleakage of three different dental biomaterials as intra-orifice barrier during nonvital bleaching. Dent Res J (Isfahan).

[26] Moradi S, Lomee M, Gharechahi M (2015). Comparison of fluid filtration and bacterial leakage techniques for evaluation of microleakage in endodontics. Dent Res J (Isfahan).

[27] Saghiri MA, Lotfi M, Saghiri AM, Vosoughhosseini S, Fatemi A, Shiezadeh V, Ranjkesh B (2008). Effect of pH on sealing ability of white mineral trioxide aggregate as a root-end filling material. J Endod.

[28] Vosoughhosseini S, Lotfi M, Shahmoradi K, Saghiri MA, Zand V, Mehdipour M, Ranjkesh B, Mokhtari H, Salemmilani A, Doosti S (2011). Microleakage comparison of glass-ionomer and white mineral trioxide aggregate used as a coronal barrier in nonvital bleaching. Med Oral Patol Oral Cir Bucal.

[29] Arslan H, Ayranci LB, Karatas E, Topcuoglu HS, Yavuz MS, Kesim B (2013). Effect of agitation of EDTA with 808-nanometer diode laser on removal of smear layer. J Endod.

